# Supplementary material for: Understanding the impact of third-party species on pairwise coexistence
Source: PLoS Comput Biol. 2022 Oct 24;18(10):e1010630. doi: 10.1371/journal.pcbi.1010630 (PMC9632822; doi:10.1371/journal.pcbi.1010630)
Supplement: S1 Appendix — (PDF) [file pcbi.1010630.s001.pdf]

S1 Appendix. Experimental communities: Fruit fly gut microbiota

The studied experimental community is compiled from Ref [1]. For the readers' convenience, below we summarize information about the species, medium, experiments, and measurements as written in Ref [1], the authors of which performed the experiments. Please refer to Ref [1] for more details.

- Host species: Wolbachia-free and virus-free Drosophila melanogaster Canton-S flies.
- Medium: 6.67% cornmeal, 2.7% active dry yeast, 1.6% sucrose, 0.75% sodium tartrate, 0.73% ethanol, 0.68% agar, 0.46% propionic acid, 0.09% methylparaben, 0.06% calcium chloride, and 0.01% molasses.
- Experimental conditions: 25 °C, 60% humidity, 12:12 h light:dark cycles, sterile conditions.
- Bacterial strains: Five fermentative lactic acid bacteria and acetic acid bacteria commonly occurring in the wild fly gut. Specifically, the five bacteria are
  - *Lactobacillus plantarum* (Lp),
  - *Lactobacillus brevis* (Lb),
  - *Acetobacter pasteurianus* (Ap),
  - *Acetobacter tropicalis* (At),
  - *Acetobacter orientalis* (Ao).
- Bacterial abundance calculations: On the 10th day of inoculation, flies were washed in 70% ethanol before being bead-beaten in 96-well plates with a custom-made attachment. Lysates were pinned onto selective media using a 96-pin replicator (Boekel), visually scored, and colony-forming units (CFUs) were enumerated.
- Bacterial combinations: 5 single bacterium, 10 pairs of bacteria, 10 triplets of bacteria, 5 quartets of bacteria, 1 quintet of all bacteria.
- Replicates: 48 replicates were performed for each bacterial combination.
- Gnotobiotic preparation: Per fly vial, each bacterium was prepared 5×10^6 CFUs (50 μL of 10^8 bacteria per milliliter) and then mixed according to the bacterial combinations. Germ-free flies were sorted into these vials.

The results of these experiments were datasets consisting of the abundances of the different bacteria species in each of the combinations and replicates after 10 days.

References

[1] Gould AL, Zhang V, Lamberti L, Jones EW, Obadia B, Korasidis N, et al. Microbiome interactions shape host fitness. Proceedings of the National Academy of Sciences. 2018;115:E11951–E11960.
