# Supplementary material for: Understanding the impact of third-party species on pairwise coexistence
Source: PLoS Comput Biol. 2022 Oct 24;18(10):e1010630. doi: 10.1371/journal.pcbi.1010630 (PMC9632822; doi:10.1371/journal.pcbi.1010630)
Supplement: S2 Appendix — (PDF) [file pcbi.1010630.s002.pdf]

S2 Appendix. Inferring the interaction matrix

To infer the pairwise interaction matrix from the fruit-fly experiments [1], we considered a subset of the above data sets containing only one or two species. Therefore, the available information indicates the abundance of species i in isolation, and how its abundance is modified when species j is included. A small (resp. large) change in the density of species i due to the inclusion of species j is evidence of a negligible (resp. significant) a_{ij}. To quantify the value of a_{ij}, let x_i^* denote the equilibrium density of the i-th species in isolation. Let (y_i^*, y_j^*) denote the equilibrium density of species i and j when both are present in the experiment (co-cultures). Then, for all data such that $x_i^* \neq 0$ and $y_i^* \neq 0$, the LV model in this form:

$$\frac{\mathrm{d}N_i}{\mathrm{d}t} = N_i \left(\theta_i - \sum_{j=1}^{S} a_{ij} N_j \right) \tag{S1}$$

implies that the following equations must be satisfied:

$$\begin{aligned} \theta_i - a_{ii} x_i^* &= 0, \\ \theta_i - a_{ii} y_i^* - a_{ij} y_j^* &= 0. \end{aligned} \tag{S2}$$

Note that, since only steady-state measurements are available, there are fundamental limitations in the information of a_{ij} that can be inferred [2]. Namely, in the above equations for each species i we have three unknown $(\theta_i, a_{ii}, a_{ij})$ but only two equations, implying there is not sufficient information to constrain the three unknowns. To circumvent this limitation, we can use the fact that the variables are homogeneous measures and any normalization will not affect the outcomes, such as assuming $\hat{a}_{ii} = 1$ [3]. With this assumption, and subtracting the first from the last line of Eq (S2), we obtain

$$a_{ij} = \frac{x_i^* - y_i^*}{y_j^*}, \tag{S3}$$

which can be used to estimate a_{ij} from the experimental data.

Importantly, since there are several replicates for x_i^* and (y_i^*, y_j^*), Eq (S3) actually characterizes a distribution of possible values for a_{ij} obtained by taking an arbitrary pair of replicates. To obtain the single value necessary for our predictions, we defined

$$\hat{a}_{ij} = \mathrm{Median}(a_{ij}), \quad i \neq j, \tag{S4}$$

estimated using a Bootstrap method with all possible 48×48 pairs. By repeating this process for all

pairs of species, we obtained the following estimate for the interaction matrix:

$$\hat{A} = \begin{array}{c} \\ \text{Lp} \\ \text{Lb} \\ \text{Ap} \\ \text{At} \\ \text{Ao} \end{array} \begin{array}{c} \begin{array}{ccccc} \text{Lp} & \text{Lb} & \text{Ap} & \text{At} & \text{Ao} \end{array} \\ \begin{pmatrix} 1 & 0 & -0.182 & -0.091 & -0.197 \\ 1.119 & 1 & 0.097 & 0.34 & 0.094 \\ -0.592 & 0 & 1 & 0 & 0 \\ 0.288 & 0.036 & 0 & 1 & 0.576 \\ 0 & 0 & 0 & 0.239 & 1 \end{pmatrix} \end{array}. \tag{S5}$$

S11 Fig shows the accuracy of this pairwise matrix in predicting the multispecies dynamics.

References

[1] Gould AL, Zhang V, Lamberti L, Jones EW, Obadia B, Korasidis N, et al. Microbiome interactions shape host fitness. Proceedings of the National Academy of Sciences. 2018;115:E11951–E11960.

[2] Angulo MT, Moreno JA, Lippner G, Barabási AL, Liu YY. Fundamental limitations of network reconstruction from temporal data. Journal of the Royal Society Interface. 2017;14(127):20160966.

[3] Xiao Y, et al. (2017) Mapping the ecological networks of microbial communities. *Nature communications* 8:1–12.
