# Supplementary material for: Understanding the impact of third-party species on pairwise coexistence
Source: PLoS Comput Biol. 2022 Oct 24;18(10):e1010630. doi: 10.1371/journal.pcbi.1010630 (PMC9632822; doi:10.1371/journal.pcbi.1010630)
Supplement: S3 Appendix — (PDF) [file pcbi.1010630.s003.pdf]

S3 Appendix. An example of constrained environmental conditions

In the main text, we assume the environmental conditions are random and heterogeneous so that the effective growth rates θ are uniformly distributed on the closed unit sphere (Fig 1). Yet, real ecological systems and experiments may suggest a specific range of environmental variation (e.g., *in vitro* laboratory experiments are typically highly-controlled), which geometrically constrains the parameter space from the entire unit sphere to a specific region. To illustrate how our framework continues to operate under these constrained scenarios, here we calculated the system-level effects for the fixed pair $\{1,2\}$ in Fig 1D within random 3-dimensional systems under constrained environments. Specifically, we assume that the effective growth rate of individual species ranges between $[-0.9, 1]$ due to environmental constraints. Fig S1 illustrates our analytical system-level effects (Fig S1A) and the projection contribution (Fig S1B) in the constrained parameter space.

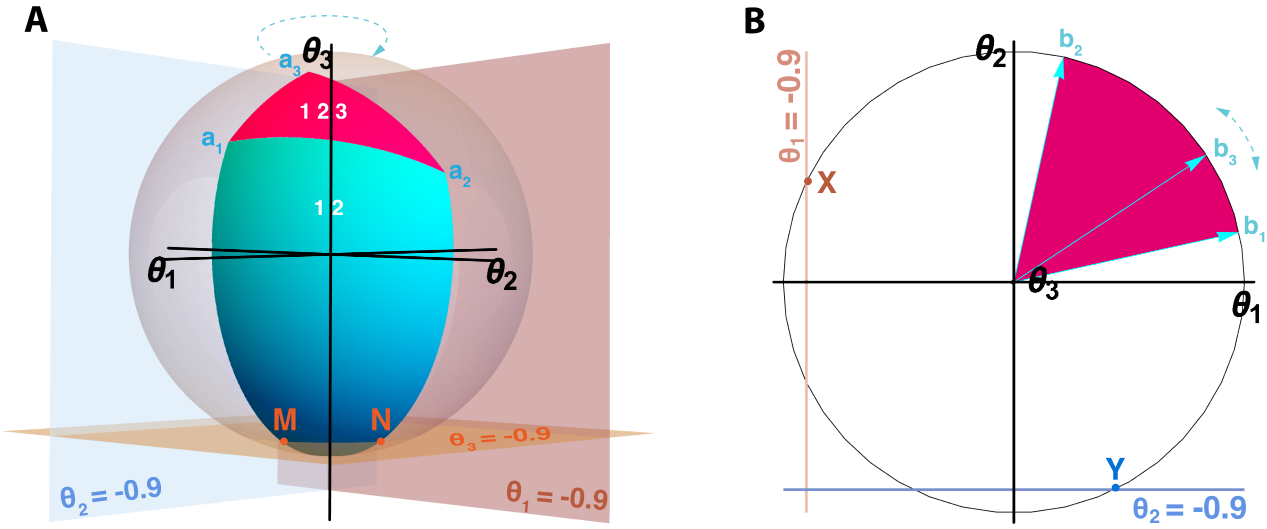

Supplementary Figure S1: **Illustration of long-term effects and projection contribution under constrained environments.** Panel **A** shows the constrained feasibility of the pair within a random 3-species system. The spanning vectors $\mathbf{a}_1, \mathbf{a}_2$ and $\mathbf{a}_3$ are the three column vectors of the interaction matrix. The parameter space is constrained by three planes $\theta_1 = -0.9, \theta_2 = -0.9$ and $\theta_3 = -0.9$. Points M and N are the intersection points of the colored feasibility region and the plane $\theta_3 = -0.9$. Panel **B** shows the constrained projection in 2-dimensional space. The spanning vectors $\mathbf{b}_1, \mathbf{b}_2$ and $\mathbf{b}_3$ are the projections of $\mathbf{a}_1, \mathbf{a}_2$ and $\mathbf{a}_3$, respectively. The parameter space is constrained by two lines $\theta_1 = -0.9$ and $\theta_2 = -0.9$. Points X and Y are two intersection points of the unit circle and the two lines.

Note that the long-term (analytical) effects are evaluated in 3-dimensional space, and the projection contribution is in 2-dimensional space. Besides, all the calculations of constrained effects can be based on the effects without constraint. The feasibility $F(\mathcal{Z})$ of the fixed pair $\mathcal{Z} = \{1,2\}$ in isolation without constraint is 0.18, and the corresponding interaction matrix is $\begin{pmatrix} 1 & 0.22 \\ 0.22 & 1 \end{pmatrix}$.

Specifically, for the long-term effects (Fig S1A), we can firstly solve the coordinates of the two intersection points: M $(0.43, 0.093, -0.9)$, N $(0.093, 0.43, -0.9)$. The plane $\theta_3 = -0.9$ removes the region

below it, whose hypervolume (i.e., surface area) is 0.093, from the feasibility regions $P(\mathcal{Z},\mathcal{S})$ of the pair $\mathcal{Z}=\{1,2\}$ within a 3-species system $\mathcal{S}$ (i.e., pink region and blue region). Besides, the three planes $\theta_1=-0.9,\ \theta_2=-0.9,\ \theta_3=-0.9$ each eliminate equal hypervolumes, which is 0.52, from the entire unit sphere. Then we have the hypervolume of the constrained parameter space $4\pi-3\times0.52=11.01$. Thus, the constrained feasibility $P'(\mathcal{Z},\mathcal{S})$ of the pair $\mathcal{Z}=\{1,2\}$ within a 3-species system $\mathcal{S}$ can be computed by

$$P'(\mathcal{Z},\mathcal{S})=\frac{P(\mathcal{Z},\mathcal{S})\times4\pi-0.093}{11.01},$$

where $P(\mathcal{Z},\mathcal{S})$ is the feasibility of the pair $\mathcal{Z}=\{1,2\}$ within a 3-species system $\mathcal{S}$ without constraint.

Similarly, the constrained feasibility $F'(\mathcal{Z})$ of the pair $\mathcal{Z}=\{1,2\}$ in isolation can be computed by

$$F'(\mathcal{Z})=\frac{F(\mathcal{Z})\times4\pi-0.093}{11.01}=\frac{0.18\times4\pi-0.093}{11.01}=0.20,$$

where $F(\mathcal{Z})$ is the feasibility of the pair $\mathcal{Z}=\{1,2\}$ in isolation without constraint.

Based on the definition, we can obtain the long-term effects of any random 3-species system $\mathcal{S}$ on pair $\mathcal{Z}=\{1,2\}$ under constrained environments by

$$LE(\mathcal{Z},\mathcal{S})=\frac{P'(\mathcal{Z},\mathcal{S})}{F'(\mathcal{Z})}.$$

For the projection contribution (Fig S1B), it is clear that the projection region of a 3-species system spanned by $\mathbf{b}_1,\ \mathbf{b}_2$ and $\mathbf{b}_3$ is always larger or equal to the feasibility region of the pair in isolation spanned by $\mathbf{b}_1$ and $\mathbf{b}_2$. The two lines $\theta_1=-0.9,\ \theta_2=-0.9$ each eliminate equal hypervolumes (arc length) from the unit circle. By the Pythagorean theorem, we can calculate the angle and then get the hypervolume 0.90 of single elimination. Thus, the constrained feasibility $F''(\mathcal{Z})$ of the pair $\mathcal{Z}=\{1,2\}$ in isolation is

$$F''(\mathcal{Z})=\frac{F(\mathcal{Z})\times2\pi}{2\pi-2\times0.90}=\frac{0.18\times2\pi}{2\pi-2\times0.90}=0.25,$$

where $F(\mathcal{Z})$ is the feasibility of the pair $\mathcal{Z}=\{1,2\}$ in isolation without constraint.

Note that the vector $\mathbf{b}_3$ corresponding to a random species can move freely along the unit circle. If the projection region is not the full two-dimensional parameter space, then its hypervolume is maximized when $\mathbf{b}_3$ moves to the point X $(-0.9,0.44)$ or Y $(0.44,-0.9)$. At these two points, the constrained projection $\mathrm{Proj}'(\mathcal{Z},\mathcal{S})$ is

$$\mathrm{Proj}'(\mathcal{Z},\mathcal{S})_{\mathrm{X}}=\mathrm{Proj}'(\mathcal{Z},\mathcal{S})_{\mathrm{Y}}=\frac{\pi-0.90/2-(\frac{\pi}{2}-F(\mathcal{Z})\times2\pi)/2}{2\pi-2\times0.90}=\frac{\pi-0.45-0.22}{2\pi-2\times0.90}=0.55.$$

Otherwise, if the projection region covers the entire constrained 2-dimensional space, then the $\text{Proj}'(\mathcal{Z}, \mathcal{S}) = 1$.

In general, when $\mathbf{b}_3$ is moving on the (longer) arc between points X and Y, the constrained projection $\text{Proj}'(\mathcal{Z}, \mathcal{S})$ can be computed by

$$\text{Proj}'(\mathcal{Z}, \mathcal{S}) = \frac{\text{Proj}(\mathcal{Z}, \mathcal{S}) \times 2\pi}{2\pi - 2 \times 0.90} = \frac{\text{Proj}(\mathcal{Z}, \mathcal{S}) \times 2\pi}{4.48} \in [0.25, 0.55] \cup \{1\},$$

where $\text{Proj}(\mathcal{Z}, \mathcal{S})$ is the projection without constraint.

Hence, according to the definition, the amount of projection contribution increasing the feasibility region in the constrained parameter space is

$$PC(\mathcal{Z}, \mathcal{S}) = \frac{\text{Proj}'(\mathcal{Z}, \mathcal{S})}{F''(\mathcal{Z})}.$$

For the short-term (simulated) effects, we sample the points on the entire unit sphere, remove the ones located outside of the constrained region, and then calculate the frequency of pairwise coexistence based on the remaining points.

The buffering effect can be easily obtained by the ratio between the short-term and long-term effects.

S6 Fig shows the distributions of aforementioned system-level effects for the fixed pair $\{1, 2\}$ within 50 different 3-dimensional systems under constrained environmental conditions. This shows that short-term effects tend to be higher (resp. lower) than long-term effects if these long-term effects are less (resp. greater) than one—confirming that our main results also hold for cases where the environmental effects are constrained to specific regions of the parameter space.
