# Supplementary material for: Understanding the impact of third-party species on pairwise coexistence
Source: PLoS Comput Biol. 2022 Oct 24;18(10):e1010630. doi: 10.1371/journal.pcbi.1010630 (PMC9632822; doi:10.1371/journal.pcbi.1010630)
Supplement: S4 Appendix — (PDF) [file pcbi.1010630.s004.pdf]

S4 Appendix. Monte Carlo estimate of measures $F(\mathcal{C}, \mathcal{S})$

One way to efficiently estimate the measure $F(\mathcal{C}, \mathcal{S})$ of a given community $\mathcal{C}$ within a larger multispecies system $\mathcal{S}$ is to use a simple Monte Carlo approach. We can randomly sample a point on the unit sphere in $S = |\mathcal{S}|$ dimensions by picking a random vector $\mathbf{x} = \sum_{i \in \mathcal{S}} \nu_i \mathbf{e}_i$ where ν_i is chosen from a Gaussian distribution with a common variance for each i. We can efficiently test whether $\mathbf{x} \in D_F(\mathcal{C}, \mathcal{S})$ by checking S linear conditions of the form $\mathbf{x} \cdot \mathbf{b} > 0$, as described below. We can then rapidly accumulate statistics on the fraction of random points in the sphere that lie in $D_F(\mathcal{C}, \mathcal{S})$ to estimate $F(\mathcal{C}, \mathcal{S})$.

In further detail, the linear conditions that determine which community $\mathcal{C} \subset \mathcal{S}$ is associated with a given point $\mathbf{x}$ can be described in terms of the boundaries between a community $\mathcal{C}$ and a community $\mathcal{C}' = \mathcal{C} \cup \{i\}, i \notin \mathcal{C}$. A necessary condition for $\mathbf{x} \in D_F(\mathcal{C}, \mathcal{S})$ or $\mathbf{x} \in D_F(\mathcal{C}', \mathcal{S})$ is that

$$(\mathsf{det}[\mathbf{a}'_{j_1} \ldots \mathbf{a}'_{j_C} \mathbf{x}]) = \mp(\mathsf{det}[\mathbf{a}'_{j_1} \ldots \mathbf{a}'_{j_C} \mathbf{e}_i]), \; j_k \in \mathcal{C}, \tag{S6}$$

where $\mathbf{a}'$ denotes the restriction of the vector $\mathbf{a}$ to the subspace associated with community $\mathcal{C}'$, and the negative/positive sign is associated with the community $\mathcal{C}/\mathcal{C}'$. This follows because the linear hypersurface that separates $\mathcal{C}, \mathcal{C}'$ is spanned by the vectors $\mathbf{a}'_{j_k}$ and $\mathbf{e_i}, i \notin \mathcal{C}'$. (Note that these conditions continue to be valid in the degenerate case $\mathcal{C} = \{\}$, which is needed to fix the boundaries of communities $\mathcal{C}' = \{i\}$ containing only a single species.) To check whether $\mathbf{x} \in D_F(\mathcal{C}, \mathcal{S})$, we thus simply check each of the S conditions associated with the boundaries of $\mathcal{C}$ with communities that differ by a single species i, where i is added or subtracted from the community depending on whether $i \in \mathcal{C}$. If all these conditions are satisfied then $\mathbf{x} \in D_F(\mathcal{C}, \mathcal{S})$.

Since each of the determinants on the LHS of Eq (S6) can be written in the form

$$\mathsf{det}[\mathbf{a}'_{j_1} \ldots \mathbf{a}'_{j_C} \mathbf{x}] = \mathbf{b}_{C,i} \cdot \mathbf{x} \,,$$

by computing the vectors $\mathbf{b}_{C,i}$ in advance for each combination C, i we can efficiently sample and test many points to give a good Monte Carlo estimate of $F(\mathcal{C}, \mathcal{S})$. A simple mathematica code implementing this Monte Carlo algorithm is available with the other code supporting the results of this work.
