# Supplementary material for: Understanding the impact of third-party species on pairwise coexistence
Source: PLoS Comput Biol. 2022 Oct 24;18(10):e1010630. doi: 10.1371/journal.pcbi.1010630 (PMC9632822; doi:10.1371/journal.pcbi.1010630)
Supplement: S5 Appendix — (PDF) [file pcbi.1010630.s005.pdf]

S5 Appendix. A simple analytic model

Simple analytic model:

In this section we briefly describe a simple model that illustrates some of the methods and results of the main paper in a simplified situation amenable to direct analytic treatment. We consider a fixed system ($\mathcal{Z}$) of two species in a larger system ($\mathcal{S}$) containing a third species where the total interaction matrix is parameterized by

$$\mathbf{A} = \begin{pmatrix} 1 & \tan\phi_1 & a \\ \tan\phi_2 & 1 & b \\ c & d & 1 \end{pmatrix} . \tag{S7}$$

In some of the following analysis we take all interactions with the third species to be small, on the order of a small parameter ϵ, so $a = \tilde{a}\epsilon, b = \tilde{b}\epsilon, etc.$, and perform a perturbative expansion in ϵ. We also set $\phi_1 = \phi_2 = \phi$ for simplicity in some of the analysis, corresponding to a symmetric interaction $a_{12} = a_{21}$.

While the focus here is on systems of three species, some of the perturbative results are directly relevant for systems with more species.

Projection contribution:

We consider the computation of the projection contribution Eq (1), governing the range of conditions (θ_1, θ_2) under which it is *possible* that species 1 and 2 can coexist (i.e., coexistence is possible for some θ_3). The angle subtended by the vectors $(1, \tan\phi), (\tan\phi, 1)$ in the 2D system is $\alpha = \pi/2 - 2\phi$, so

$$F(\mathcal{Z}) = \frac{\alpha}{2\pi} = 1/4 - \phi/\pi \,.$$

The projection region $D_{\text{proj}}(\mathcal{Z}, \mathcal{S})$ only depends upon a, b. Parameterizing $a = \epsilon\cos\eta, b = \epsilon\sin\eta$, from simple geometry we can compute the projection fraction

$$\text{Proj}(\mathcal{Z}, \mathcal{S}) = \begin{cases} 1/4 - (\eta + \pi)/2, & -\pi/2 - \phi \le \eta \le \phi \\ 1/4 - \phi/\pi, & \phi \le \eta \le \pi/2 - \phi \\ (\eta - \phi)/2\pi, & \pi/2 - \phi \le \eta \le \pi + \phi \\ 1, & \pi + \phi \le \eta \le 3\pi/2 - \phi \end{cases}$$

Thus, we see that for random a, b the distribution of the projection fraction is such that $\text{Proj} = 1/4 - \phi/\pi$ with probability $1/4 - \phi/\pi$, $\text{Proj} = 1$ with probability $1/4 - \phi/\pi$, and uniformly distributed between the minimum value and 1/2 in the remaining cases of total probability $1/2 + 2\phi/\pi$. The projection contribution PC arises after dividing this by the minimum projection value $1/4 - \phi/\pi$. This matches qualitatively with, e.g., Fig 1D, where there are similar contributions to PC at 1 and $1/F(\mathcal{Z})$, and a roughly uniform distribution between 1 and $1/2F(\mathcal{Z})$. Note that this analysis is valid for arbitrary ϵ, not necessarily small.

Long-term system effects

We now consider the long-term (analytic) effect governing the *probability* that the species 1, 2 can coexist for randomly chosen environmental conditions $\theta_1, \theta_2, \theta_3$. This probability is given by the union of the feasibility regions $P(\mathcal{Z}, \mathcal{S}) = F(\{1,2\}, \mathcal{S}) + F(\{1,2,3\}, \mathcal{S})$. We can analyze this analytically using an analytic formula for the solid angle Ω of a triangle bounded by three rays $\mathbf{x}, \mathbf{y}, \mathbf{z}$

$$\tan(\Omega) = \mathsf{det}(\mathbf{xyz})/(xyz + (\mathbf{x}\cdot\mathbf{y})z + (\mathbf{x}\cdot\mathbf{z})y + (\mathbf{y}\cdot\mathbf{z})x)\,.$$

To leading order in ϵ, for fixed a, b, c, d of order 1, we have

$$P(\mathcal{Z}, \mathcal{S}) = \frac{1}{4\pi}\left(2\alpha - (a+b)(\cos\phi - \sin\phi) + \mathcal{O}(\epsilon^2)\right) = F(\mathcal{Z})(1 - (a+b)(1 - \alpha^2/24 + \mathcal{O}(\alpha^4))/2\sqrt{2})\,,$$

so to this order the long-term effect is $\mathrm{LE}(\mathcal{Z}, \mathcal{S}) = 1 - (a+b)/2\sqrt{2} + \mathcal{O}(\epsilon^2, \alpha^2)$ for small angles α, as can be seen naturally from the geometry. On the other hand, separating the parameters ϕ_1, ϕ_2 and taking both to be small of order ϵ, we have $\alpha \sim \pi/2 - \phi_1 - \phi_2$, so $\mathrm{F}(\mathcal{Z}) \sim 1/4 - (\phi_1 + \phi_2)/\pi$, and

$$\mathrm{LE}(\mathcal{Z}, \mathcal{S}) = 1 - (a+b)/\pi + \mathcal{O}(\epsilon^2) \sim 1 - 0.318(a+b)$$

This shows that to leading order for a symmetric probability distribution on $a + b$ around 0, the distribution on LE is symmetric and centered around 1, compatible with, e.g., Fig 1D. A more careful analysis gives the second order effect

$$F(\mathcal{Z}, \mathcal{S}) = 2\alpha - (a+b)(\cos\phi - \sin\phi) + \frac{1}{2}\left(bc + ad - (ac + bd)\cos\alpha + (2ab + bc + ad)\sin\alpha\right) + \mathcal{O}(\epsilon^3)\,. \tag{S8}$$

Since there are no quadratic terms in any of the coefficients a, b, c, d, in a random distribution where these coefficients are uncorrelated there is also no second order shift in the mean of the LE distribution. All cubic terms are odd in at least one variable so also do not shift the mean in a symmetric distribution. There are, however, quartic terms, such as $-a^4 \cos\alpha \sin\alpha/8$ that contribute to a shift of the mean. This gives some indication, however, of the reason why the mean of LE is so close to 0, so that the presence of a random environment has on average negligible effect on the probability of coexistence of a fixed pair.

Note that the linear effect of small interactions of a third species on LE for a given pair of species carries over to larger numbers of additional species; the linear effect on LE of a set of $|\mathcal{S}| - 2$ additional species on a given pair is simply given by the sum of the linear effects from each individual additional species. Thus, to leading order, $\mathrm{LE}(\mathcal{Z}, \mathcal{S}) = \prod_{i=3}^{|\mathcal{S}|} \mathrm{LE}(\mathcal{Z}, \mathcal{Z} \cup \{i\})$.

Short-term effects

A detailed analysis of the short-term effects associated with changes in the time of persistence of the pair in the larger community is somewhat more subtle, even in this simple analytic model, as it involves dynamics. To get a simple sense of the order of magnitude of this effect, however, we can consider the dynamical equations such as

$$\dot{N}_1 = N_1(\theta_1 - N_1 - \tan\phi_1 N_2 - aN_3) \tag{S9}$$

from the interaction matrix Eq (S7), where in the analysis of this section we take ϕ_1, ϕ_2 small so $\tan\phi_i \sim \phi_i$. We set all the parameters in Eq (S7) to vanish except a and focus on the effect of a small interaction term of this type; the parameters other than a and the equivalent parameter b do not have effects at linear order. For a fixed threshold of extinction, we can associate the extinction boundary in θ_1 with a certain effective negative growth rate

$$\theta_1 - N_1 - aN_3 < \text{constant}\,. \tag{S10}$$

The change in this boundary value of θ_1 under the perturbation a will be of order $aN_3 \sim a\theta_3$. Thus, when the extinction threshold η is fixed and very small and the time of simulation T is long enough that the extinction boundary is near the coexistence boundary ($T \gg |\ln\eta|$), the region in the θ-sphere where the extinction condition on the pair in isolation and in the larger set differ will be near the boundary $\theta_1 = 0$ and will have an area of roughly $a\int_0^{\pi/2}\sin\theta = a$. Thus we have roughly

$$\text{SE}(\mathcal{Z}, \mathcal{S}) \sim 1 - a/\pi \tag{S11}$$

From this we see that in the limit where the cutoff is small and the simulation time is large, BE $\to 1$. On the other hand, for larger cutoff η and shorter time scales T, the extinction boundary occurs at some fixed negative $\theta_1 \sim (\ln\eta)/T$ (with initial effects shifting $\ln\eta, T$ by small finite constants), and the difference in area will go as roughly $a(1-\theta_1^2/2)$, so we expect an effect something like

$$\text{BE}(\mathcal{Z}, \mathcal{S}) = \text{SE}(\mathcal{Z}, \mathcal{S})/\text{LE}(\mathcal{Z}, \mathcal{S}) \sim 1 + a(\ln\eta)^2/(2t^2)\,. \tag{S12}$$

An example of this is shown in Fig S2. Thus, the perturbative analysis suggests that for reasonably short time scales and appropriate cutoffs, the linearized effect will lead to BE being positive (negative) when AE is negative (positive), as seen in many of the other examples in the paper. Nonlinear effects, however, will play an important role in multi-species systems with strong interactions, and the detailed relationship in general between BE and AE depends on the precise cutoff and time scale used to compute SE and appears to be relatively complicated for systems outside the perturbative regime.

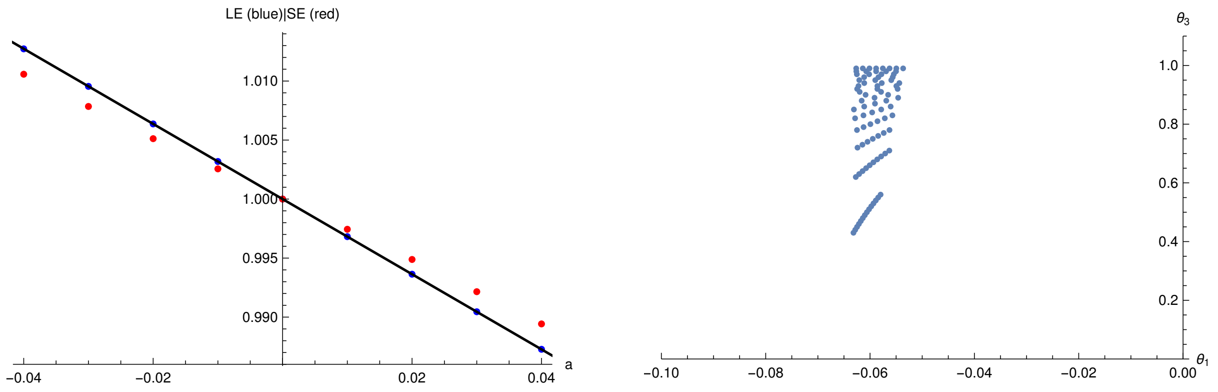

Supplementary Figure S2: **Long-term and short-term effects in the perturbative regime.** The effect of a third species on a pair of species (1, 2) with small interaction parameter $a \neq 0$ and other interactions vanishing in Eq (S7). In Panel (A), black line is the perturbative theoretical prediction for LE Eq (S8), and blue dots are exact analytic values of LE. Red dots are simulated effects SE, for extinction threshold $\eta = 0.0001$ and time $T = 100$, using 125,000 environmental conditions θ uniformly distributed on the unit sphere and initial conditions with all populations at 0.5. This example illustrates the general trend that LE > 1 (< 1) is correlated with BE = SE/LE < 1 (> 1). Panel (B) depicts the set of initial conditions where the persistence of species (1, 2) differs in the presence or absence of the third species (3) over the finite time simulations, in the case $a = 0.01$, illustrating the shape expected from the theoretical analysis. Detailed pattern of points reflects sampling choice over sphere (grid points uniformly spaced in $\theta_3, \tan^{-1}(\theta_2/\theta_1)$); extinction boundary is localized near $\theta_1 \sim -0.06 \sim (\ln \eta)/T$ (up to finite shift of $\ln \eta, T$ from initial conditions), and expands in width roughly as θ_3 as predicted.
