# Supplementary figures and images for: Understanding the impact of third-party species on pairwise coexistence
Source: PLoS Comput Biol. 2022 Oct 24;18(10):e1010630. doi: 10.1371/journal.pcbi.1010630 (PMC9632822; doi:10.1371/journal.pcbi.1010630)

**A**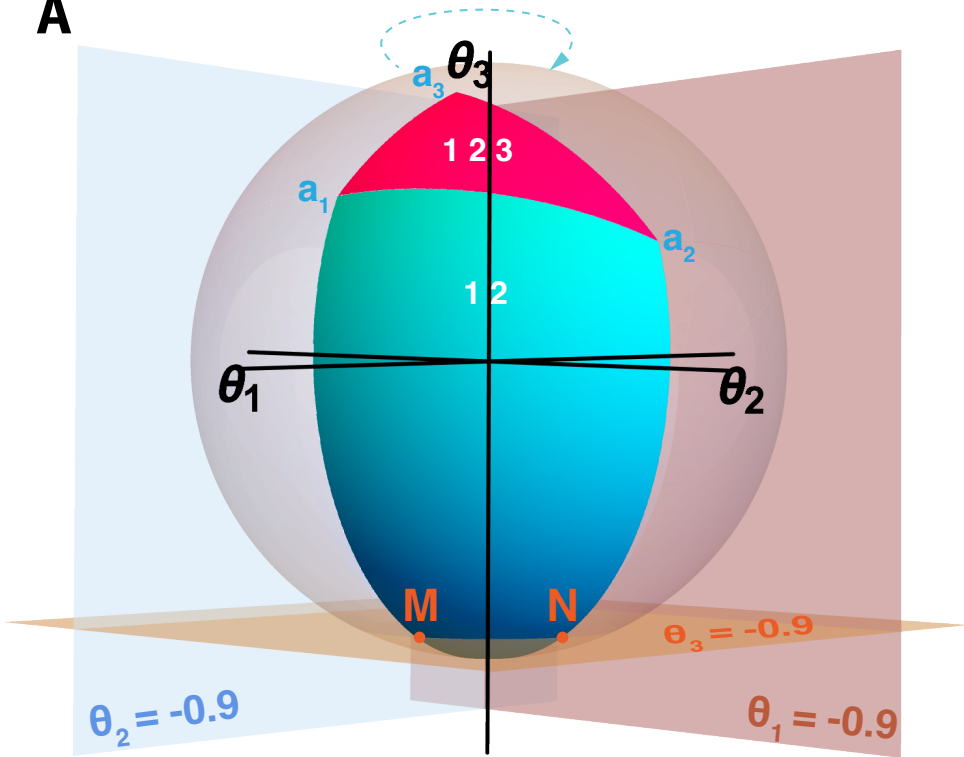**B**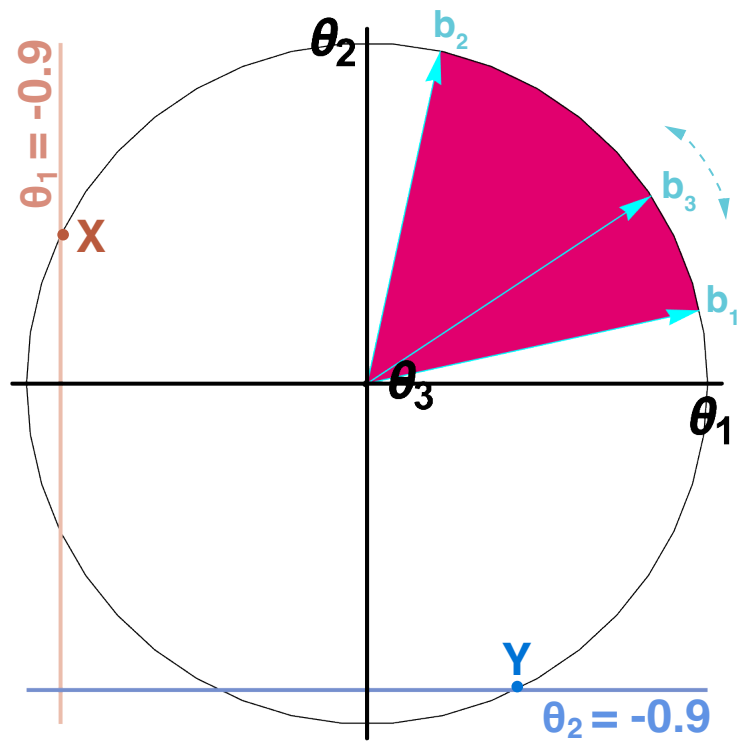

Supplement: S1 Fig — Panel A shows the constrained feasibility of the pair within a random 3-species system. The spanning vectors a1, a2 and a3 are the three column vectors of the interaction matrix. The parameter space is constrained by three planes θ1 = −0.9, θ2 = −0.9 and θ3 = −0.9. Points M and N are the intersection points of the colored feasibility region and the plane θ3 = −0.9. Panel B shows the constrained projection in 2-dimensional space. The spanning vectors b1, b2 and b3 are the projections of a1, a2 and a3, respectively. The parameter space is constrained by two lines θ1 = −0.9 and θ2 = −0.9. Points X and Y are two intersection points of the unit circle and the two lines. S6 Fig shows the distributions of system-level effects for the fixed pair {1, 2} within 50 different 3-species systems under constrained environmental conditions. Please refer to S3 Appendix for more details. (PDF) [file pcbi.1010630.s006.pdf]

LE (blue)|SE (red)

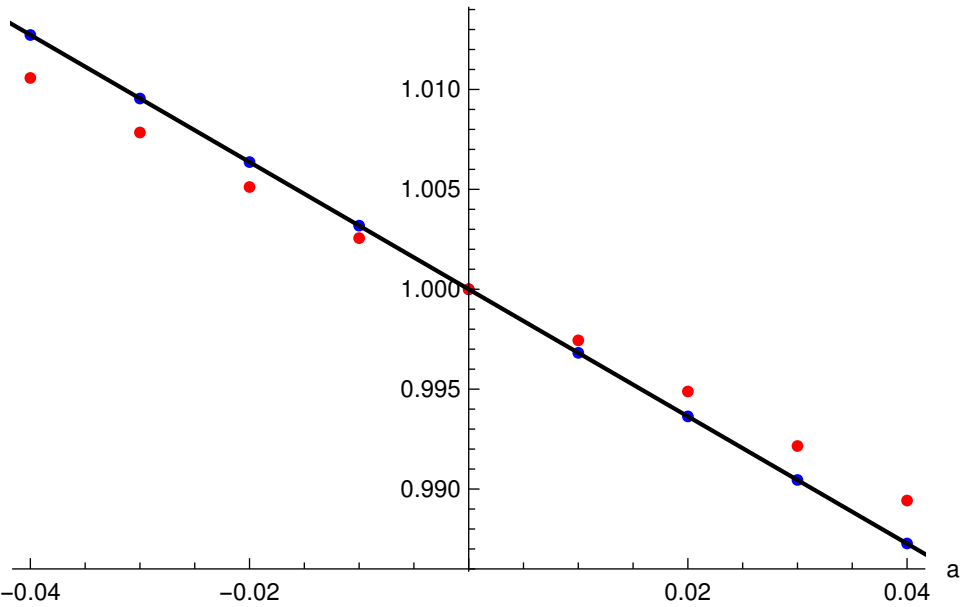

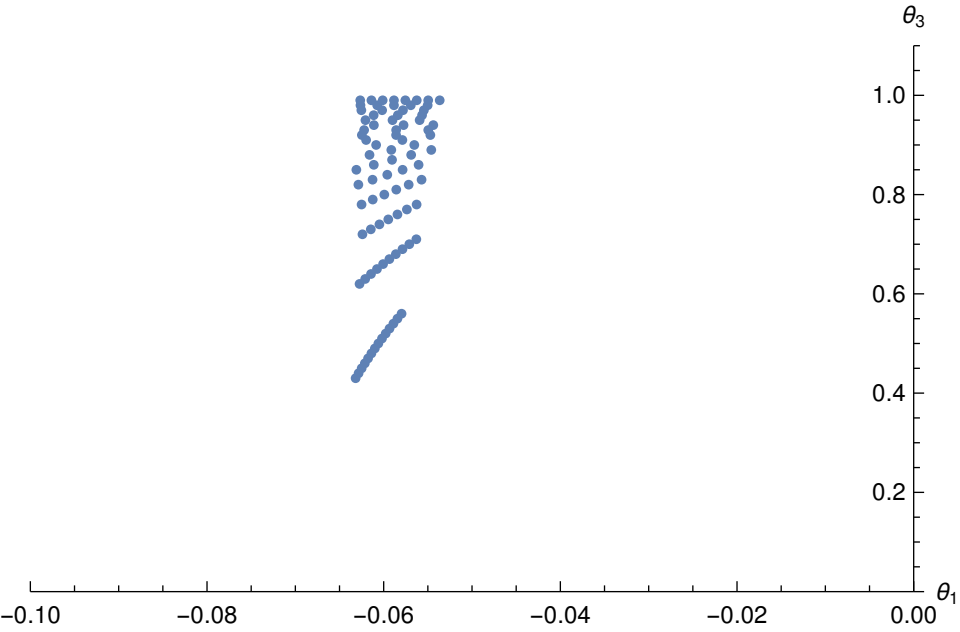

Supplement: S2 Fig — The effect of a third species on a pair of species {1, 2} with small interaction parameter a ≠ 0 and other interactions vanishing in Eq (S7) in S5 Appendix. In Panel (A), black line is the perturbative theoretical prediction for the long-term effects (LE) Eq (S8) in S5 Appendix, and blue dots are exact analytic values of LE. Red dots are simulated short-term effects (SE), for extinction threshold η = 0.0001 and time T = 100, using 125,000 environmental conditions θ uniformly distributed on the unit sphere and initial conditions with all populations at 0.5. This example illustrates the general trend that LE >1 (<1) is correlated with the buffering effects BE = SE/LE <1 (>1). Panel (B) depicts the set of initial conditions where the persistence of species {1, 2} differs in the presence or absence of the third species {3} over the finite time simulations, in the case a = 0.01, illustrating the shape expected from the theoretical analysis. Detailed pattern of points reflects sampling choice over sphere (grid points uniformly spaced in θ3, tan−1(θ2/θ1)); extinction boundary is localized near θ1 ∼ −0.06 ∼ (ln η)/T (up to finite shift of ln η, T from initial conditions), and expands in width roughly as θ3 as predicted. Please refer to S5 Appendix for more details. (PDF) [file pcbi.1010630.s007.pdf]

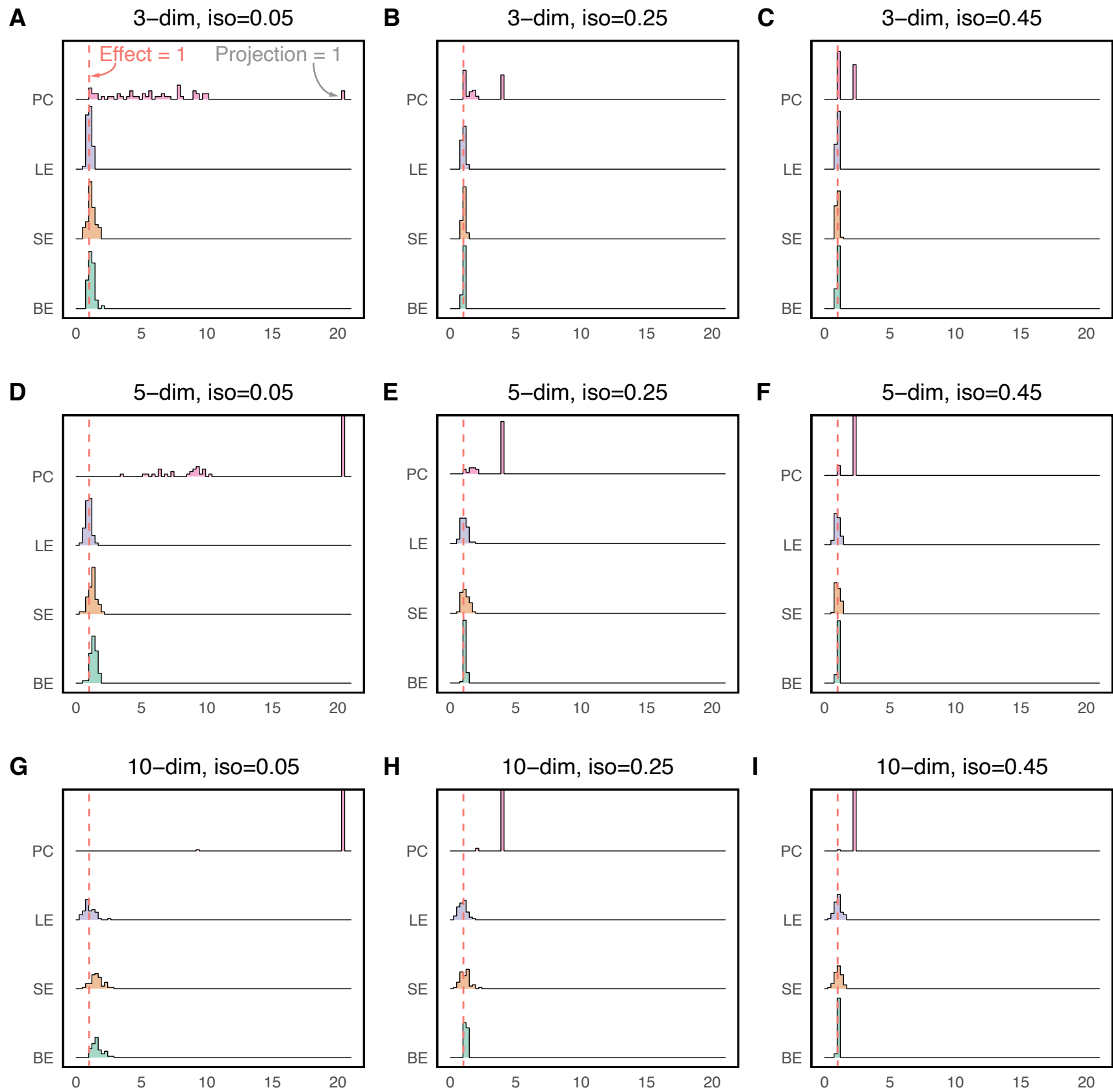

Supplement: S3 Fig — PC means projection contribution; LE means long-term effects; SE means short-term effects; BE means buffering effects. In Panels A, B, C, the 50 random systems are 3-dimensional; in Panels D, E, F, the 50 random systems are 5-dimensional; in Panels G, H, I, the 50 random systems are 10-dimensional. In Panels A, D, G, the feasibility of the fixed pair in isolation (iso) is 0.05; in Panels B, E, H, the feasibility of the fixed pair in isolation is 0.25; in Panels C, F, I, the feasibility of the fixed pair in isolation is 0.45. For reference, the dashed line shows the value of one: the relative feasibility in isolation. The conclusions are summarized under S5 Fig where the sampling distribution of the interactions associated with other species in the systems has a standard deviation of 1 (i.e., σ = 1). (PDF) [file pcbi.1010630.s008.pdf]

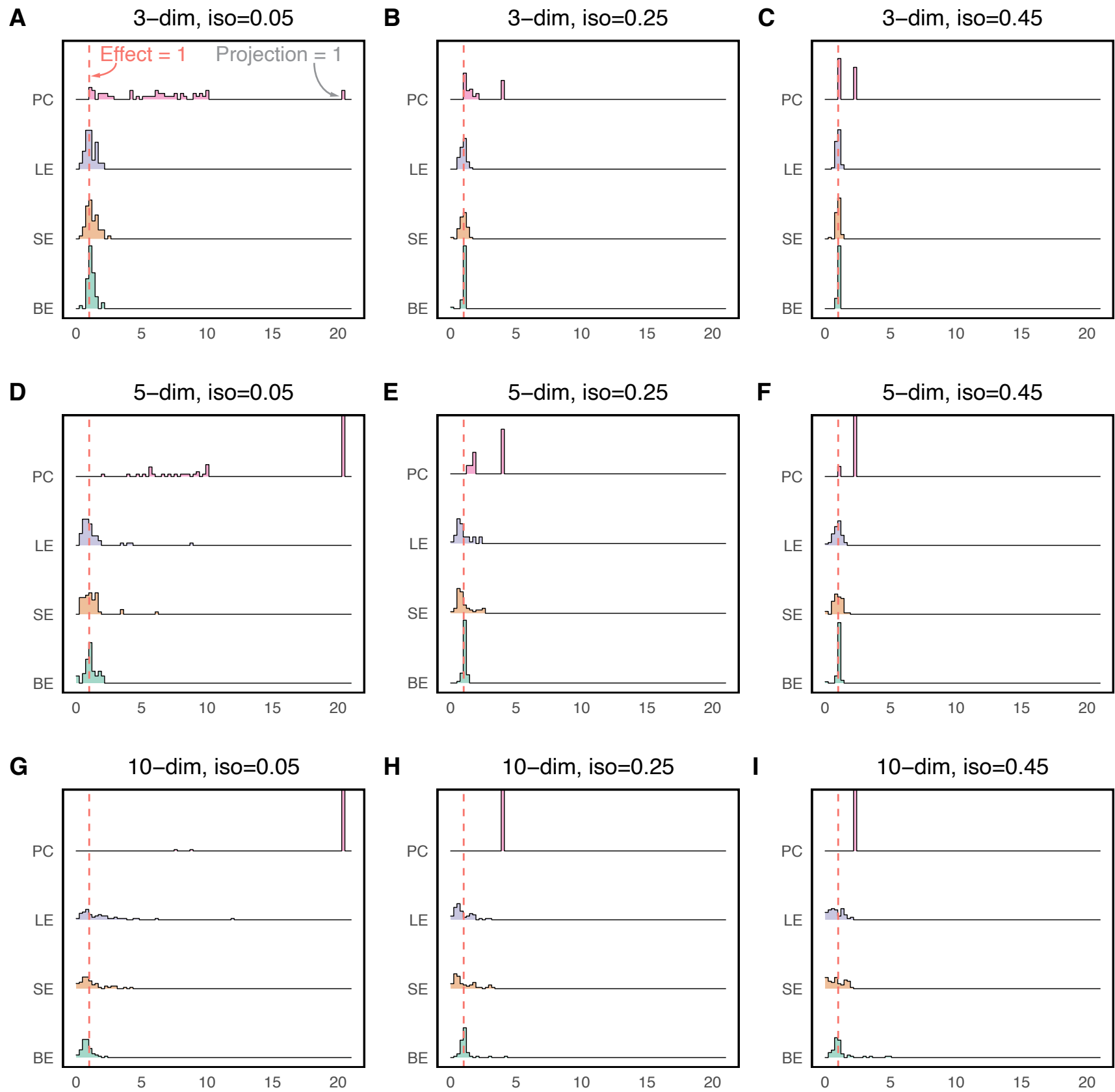

Supplement: S4 Fig — The legend and layout of panels are the same as S3 Fig. The conclusions are summarized under S5 Fig where the sampling distribution of the interactions associated with other species in the systems has a standard deviation of 1 (i.e., σ = 1). (PDF) [file pcbi.1010630.s009.pdf]

**A**

3-dim, iso=0.05

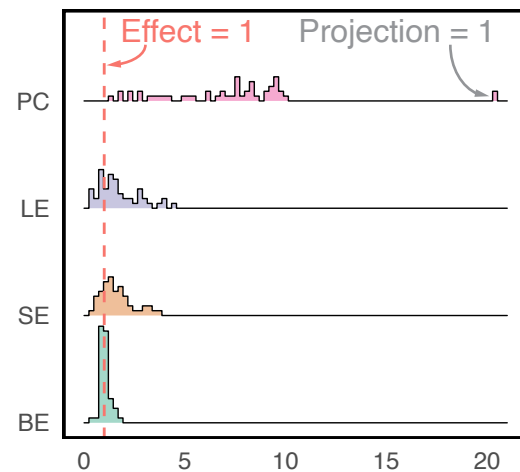**B**

3-dim, iso=0.25

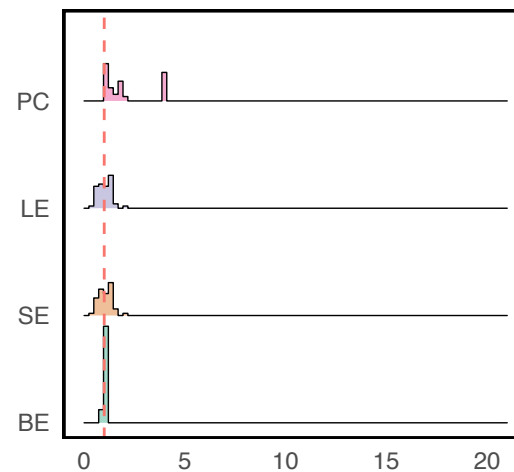**C**

3-dim, iso=0.45

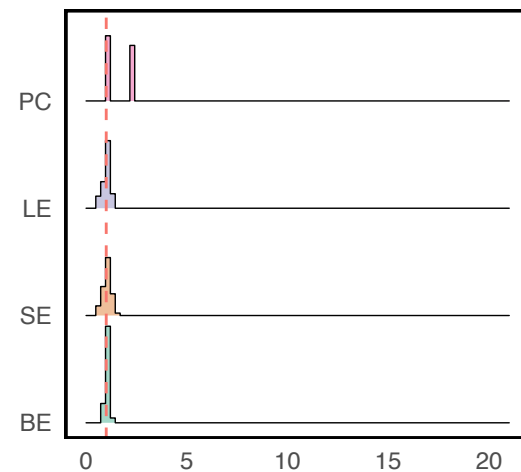**D**

5-dim, iso=0.05

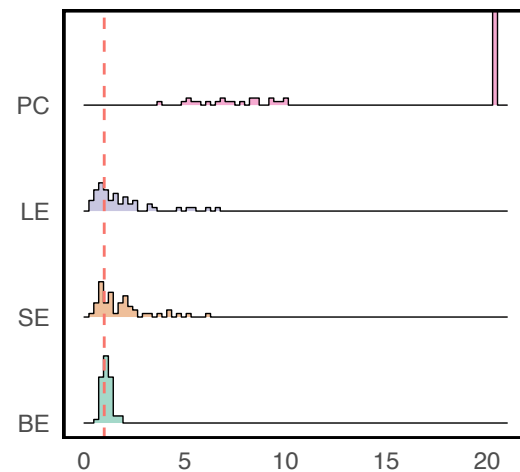**E**

5-dim, iso=0.25

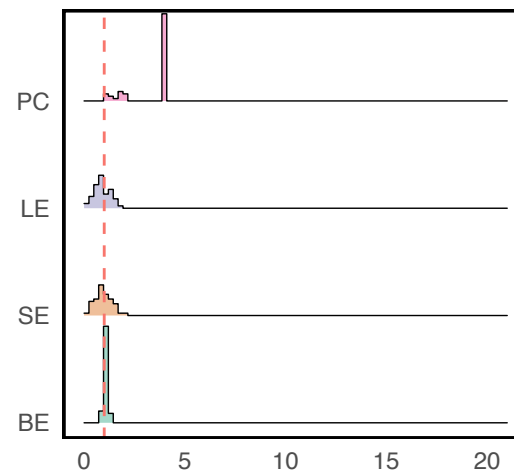**F**

5-dim, iso=0.45

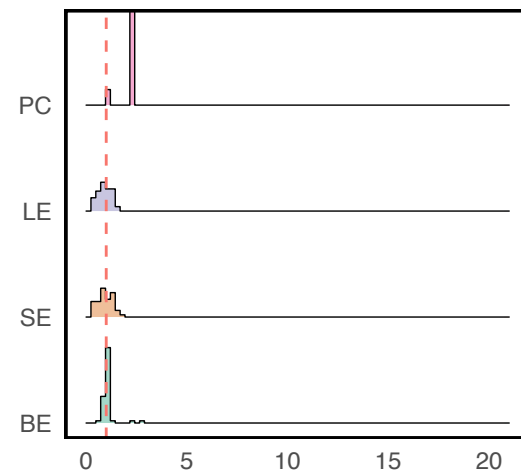

Supplement: S5 Fig — The legend and layout of panels are the same as S3 Fig. The following conclusions are based on S3, S4, and S5 Figs. The distribution of projection contribution (PC) clusters at the maximum (when projection = 1) as the dimension of systems (dim) increases. The range of PC (width of distribution) decreases as the feasibility of the pair in isolation (iso) increases. The distributions of long-term effects (LE), short-term effects (SE), and buffering effects (BE) tend to have larger widths as either the dimension of systems (dim) or the standard deviation of the sampling distribution of interactions (σ) increases, or as the feasibility of the pair in isolation (iso) decreases. According to statistical tests at the 0.05 level of significance, the distribution of long-term effects (LE) is centered at 1 and the mean of buffering effects (BE) is larger than 1 (except for one case in Panel G of S4 Fig). (PDF) [file pcbi.1010630.s010.pdf]

# A pair in random 3-species systems under constrained environmental conditions

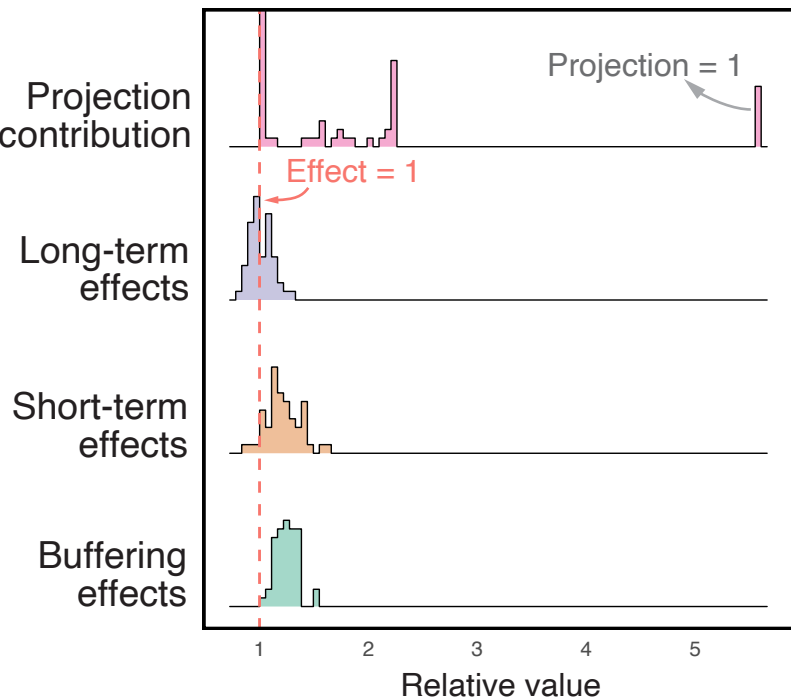

Supplement: S6 Fig — In the main text, we assume that the heterogeneous environments are completely unknown, so the effective growth rates θ of all species in the systems are uniformly distributed on the unit sphere (Fig 1). Under environmental constraints, here we assume that the growth rate of individual species ranges between [−0.9, 1]. Each point in the distributions corresponds to the same pair in a different system. As a reference, the interaction matrix of the pair in isolation is (10.220.221). (PDF) [file pcbi.1010630.s011.pdf]

A

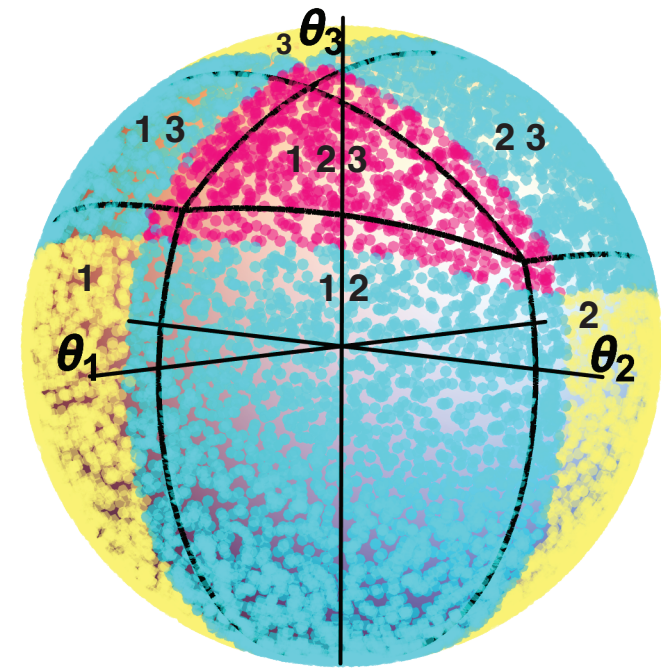

B

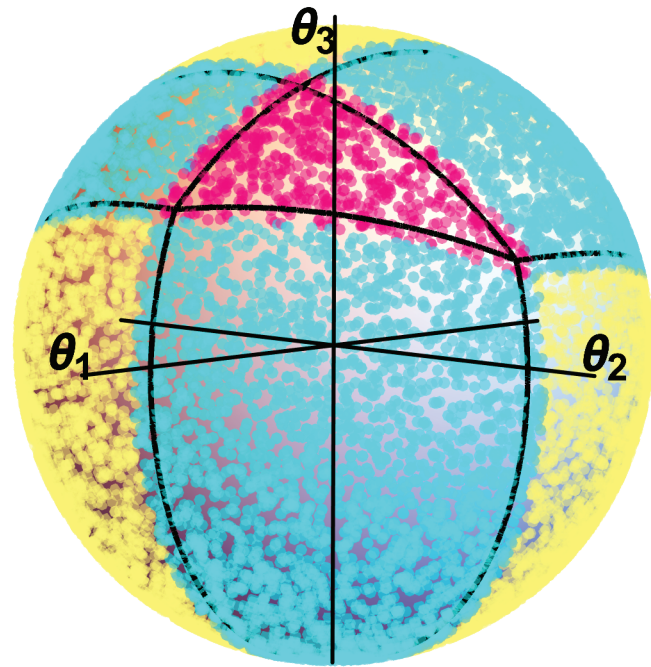

C

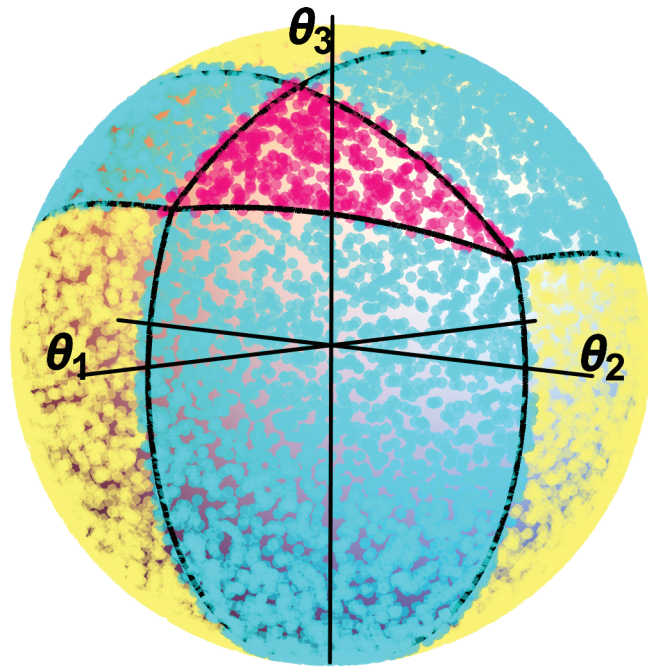

D

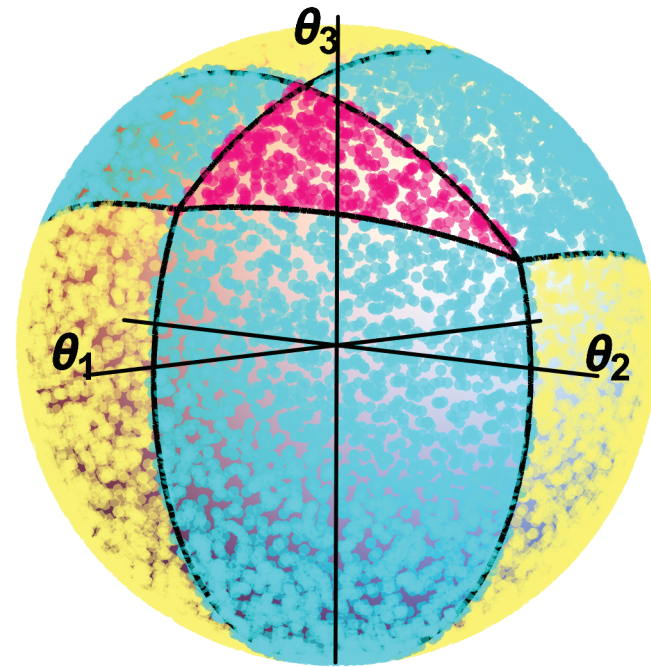

Simulation time increasing

Supplement: S7 Fig — The total time of simulations in Panels A, B, C, and D are 100, 200, 400, and 800, respectively. The step size is fixed as 0.01. The simulations are conducted by the Runge-Kutta method. The black lines correspond to the borders of the analytical feasibility regions in Fig 1B. As time increases, the points on the sphere tend to cluster in the corresponding analytical feasibility regions. As a reference, the interaction matrix of the 3-species system is (10.220.560.2210.370.560.371). (PDF) [file pcbi.1010630.s012.pdf]

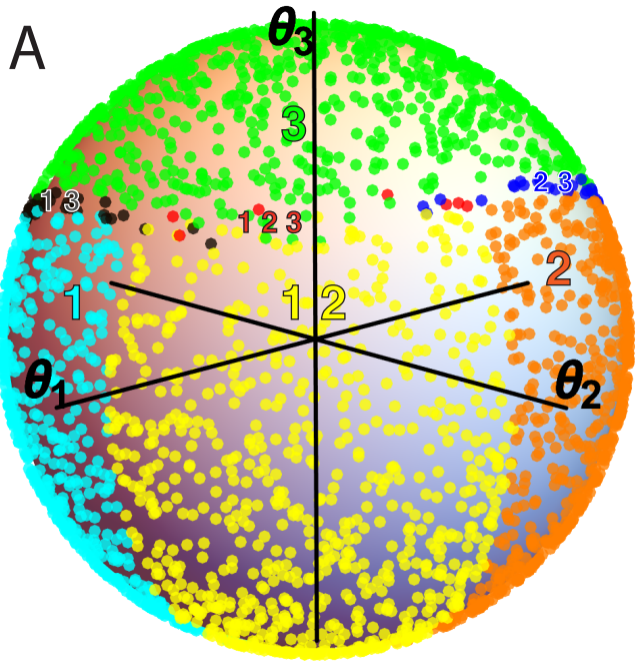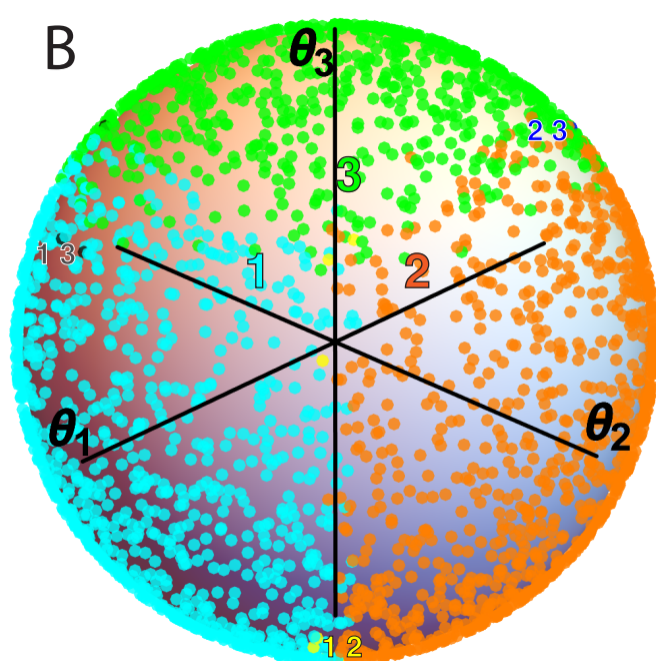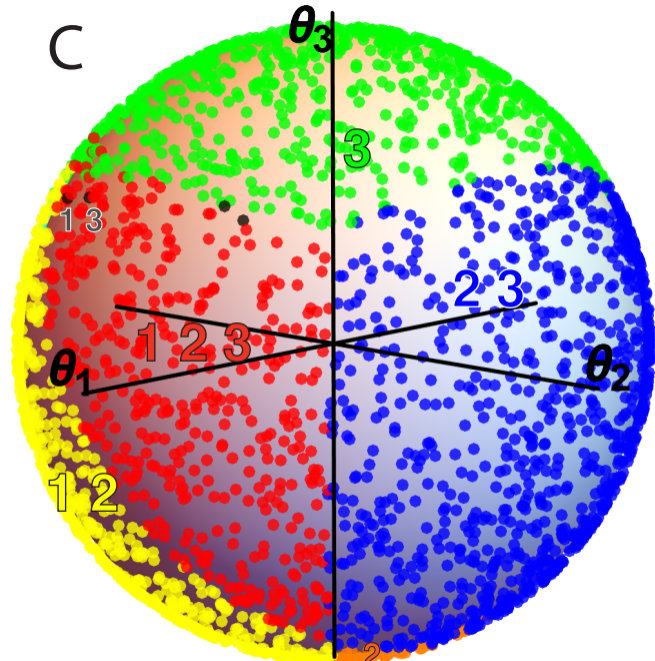

Supplement: S8 Fig — In Panel A, species 3 has strong harmful effects on species 1 and 2. In Panel B, all three species have strong harmful effects on each other. In Panel C, species have either strong harmful or strong beneficial effects on each other. In the numerical settings of all cases, the total time is 200, step size is 0.01 and extinction threshold is 10−6. The simulations are conducted by the Runge-Kutta method. As a reference, the interaction matrices in Panels A, B, and C are (10.221.560.2211.370.70.81), (11.221.561.2211.371.71.81), and (11.221.56-1.2211.371.7-1.81), respectively. Also, the three systems here are not globally stable. (PDF) [file pcbi.1010630.s013.pdf]

# Fruit fly gut microbiota

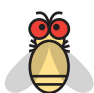

**A**

250 simulations

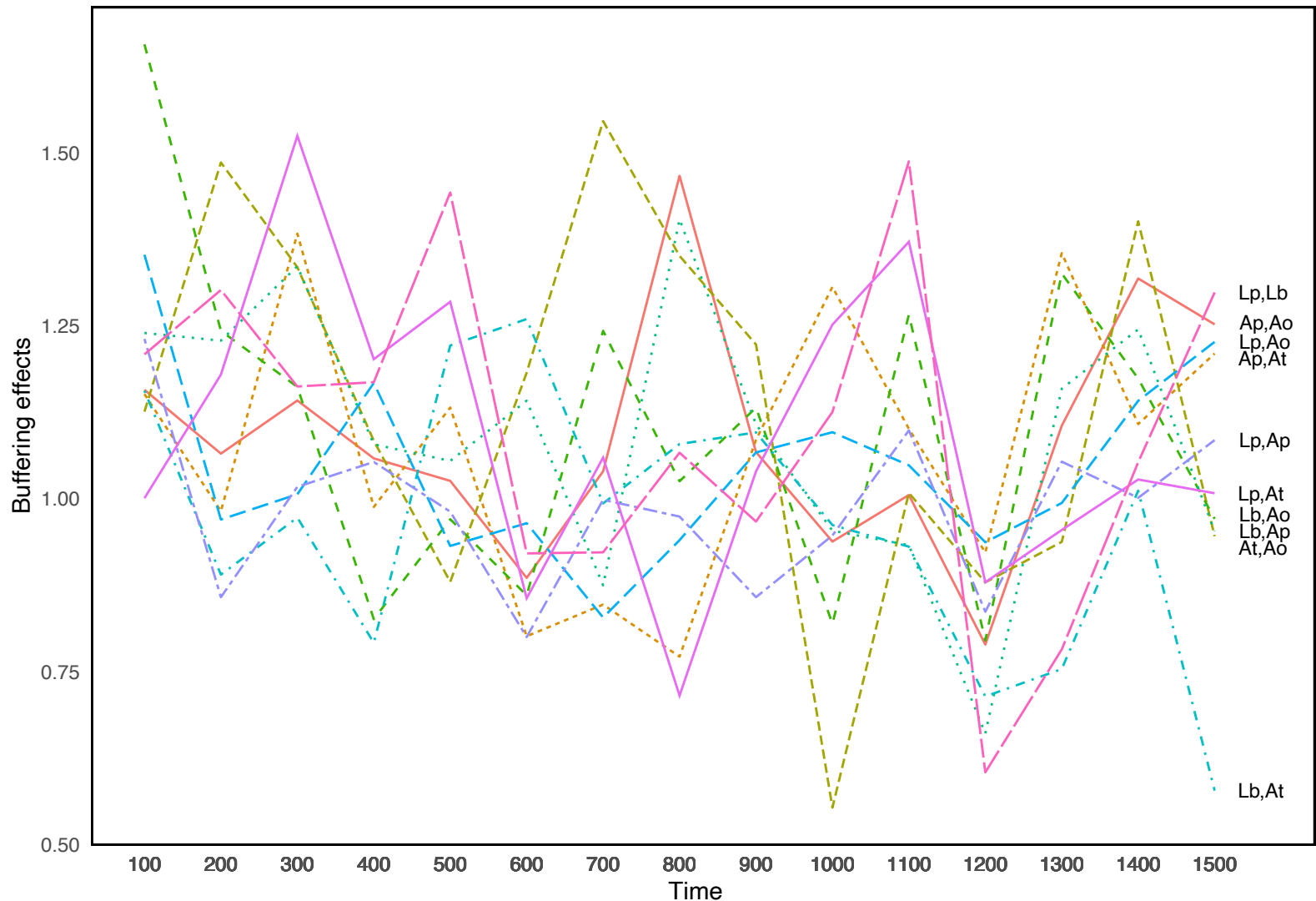

**B**

3000 simulations

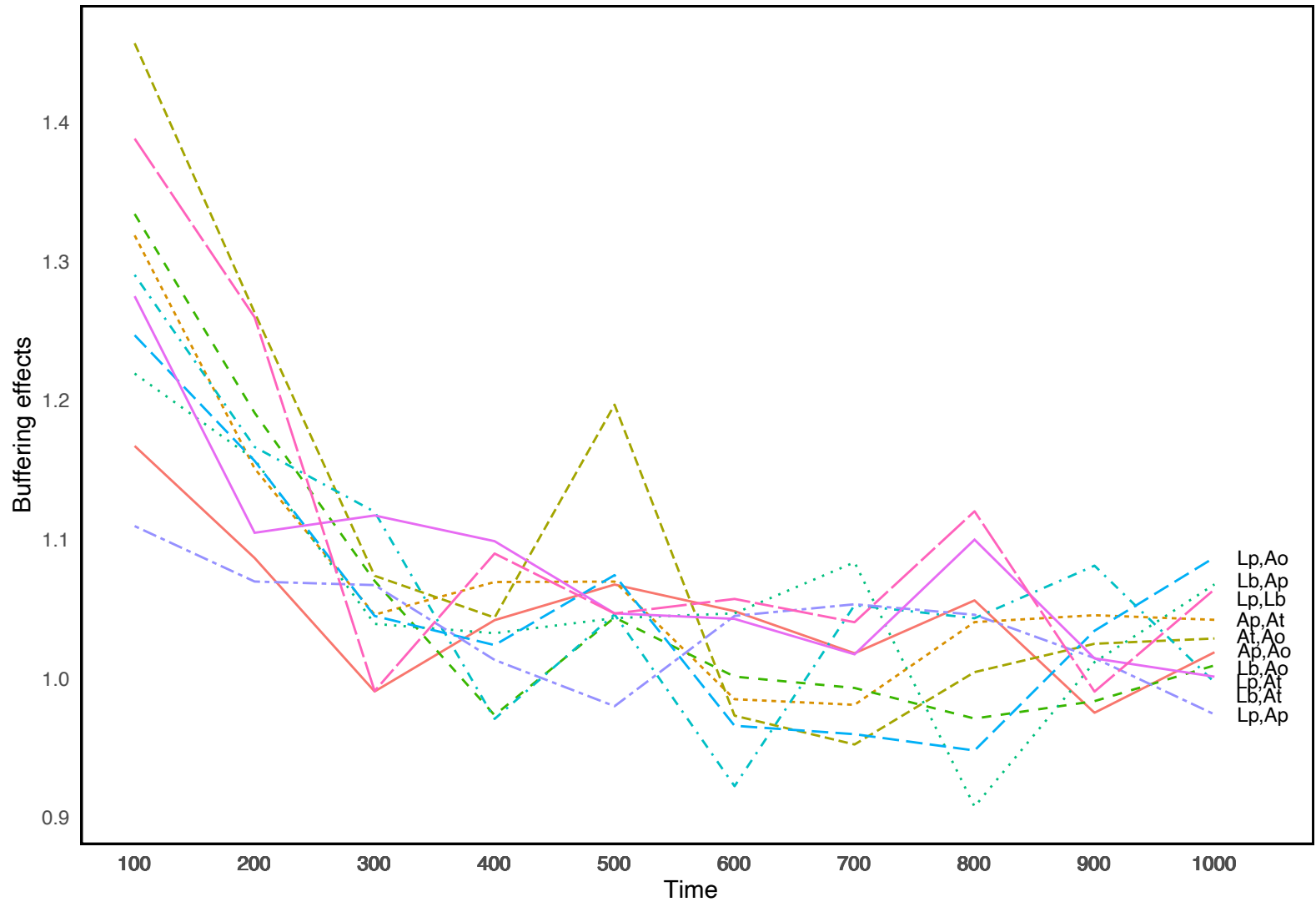

Supplement: S10 Fig — As time increases, the distribution of buffering effects (y-axis) of the system on pairwise coexistence gradually shifts to a position centered at zero. The shift is faster and smoother as the number of simulations increases (Panel A: 250 simulations; Panel B: 3000 simulations). In the numerical settings of both cases, the size of time step (x-axis) is fixed as 0.01 and the extinction threshold is 10−6. Here, the interaction matrix of fruit-fly experiments is inferred as shown in S2 Appendix. (PDF) [file pcbi.1010630.s015.pdf]

# Fruit fly gut microbiota

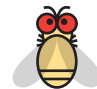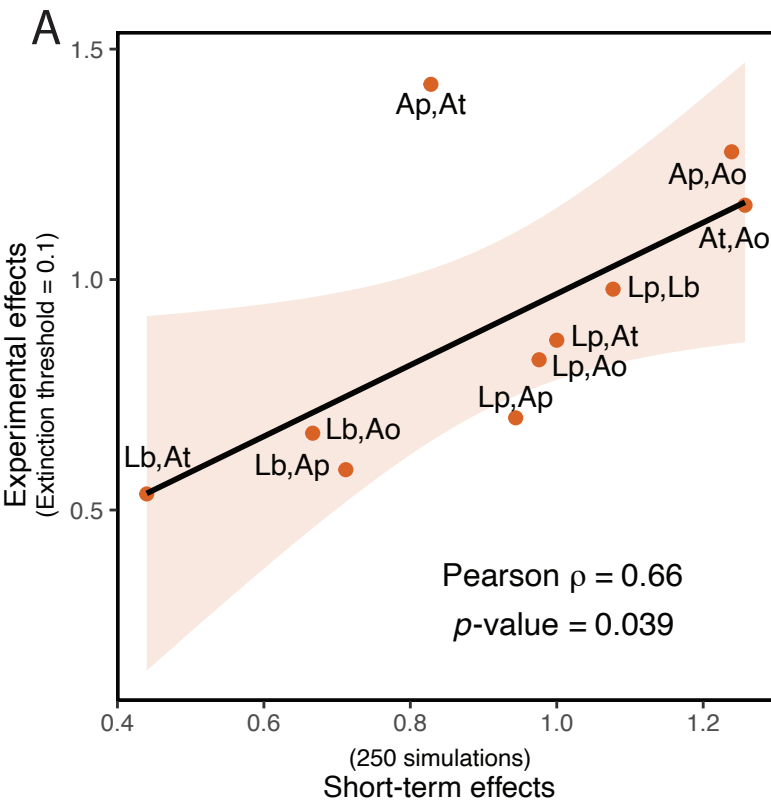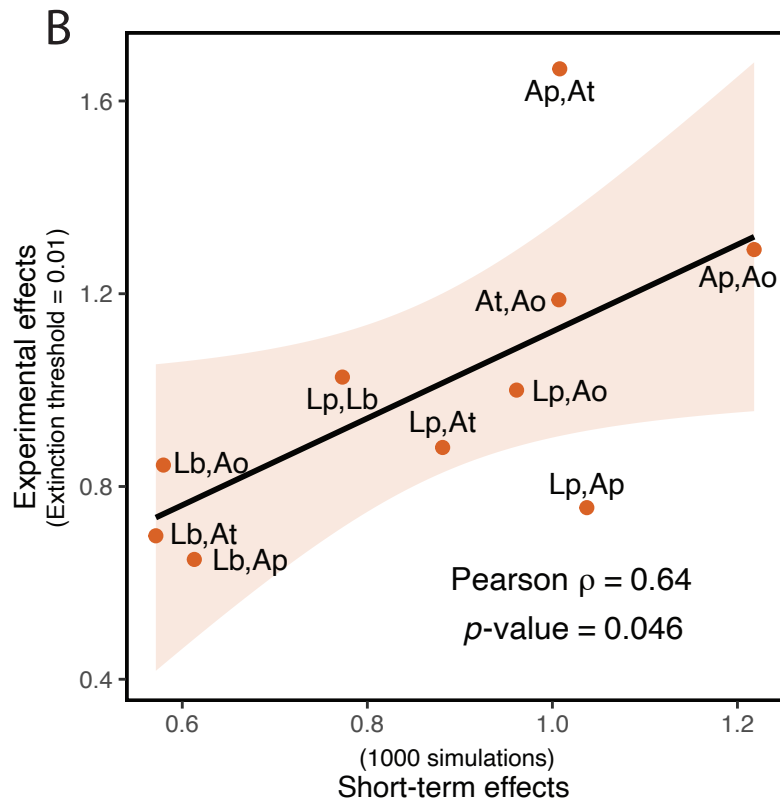

Supplement: S11 Fig — When calculating the experimental effects (y-axis), we classify a species statistically extinct in a trial if its relative abundance was less than 1% (Panel A) and 10% (Panel B), respectively, across all replicates. The short-term effects (x-axis) are obtained by simulating the Lotka-Volterra dynamics using our inferred interaction matrix in S2 Appendix for 250 times (Panel A) and 1000 times (Panel B), respectively. In the numerical settings of both cases, the total time is 200 with step size 0.01 and the extinction threshold is 10−6. The simulations are conducted by the Runge-Kutta method. We found strong Pearson correlations (Panel A: ρ = 0.66, p − value = 0.039; Panel B: ρ = 0.64, p − value = 0.046) between the experimental effects and the short-term effects. Thus, with suitable parameters (e.g. number of simulations, extinction threshold, simulation runtime), the numerical simulations can successfully capture the information of pairwise coexistence within multispecies systems in the experimental settings. (PDF) [file pcbi.1010630.s016.pdf]

# Fruit fly gut microbiota

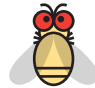

(Extinction threshold in the experimental settings = 0.01)

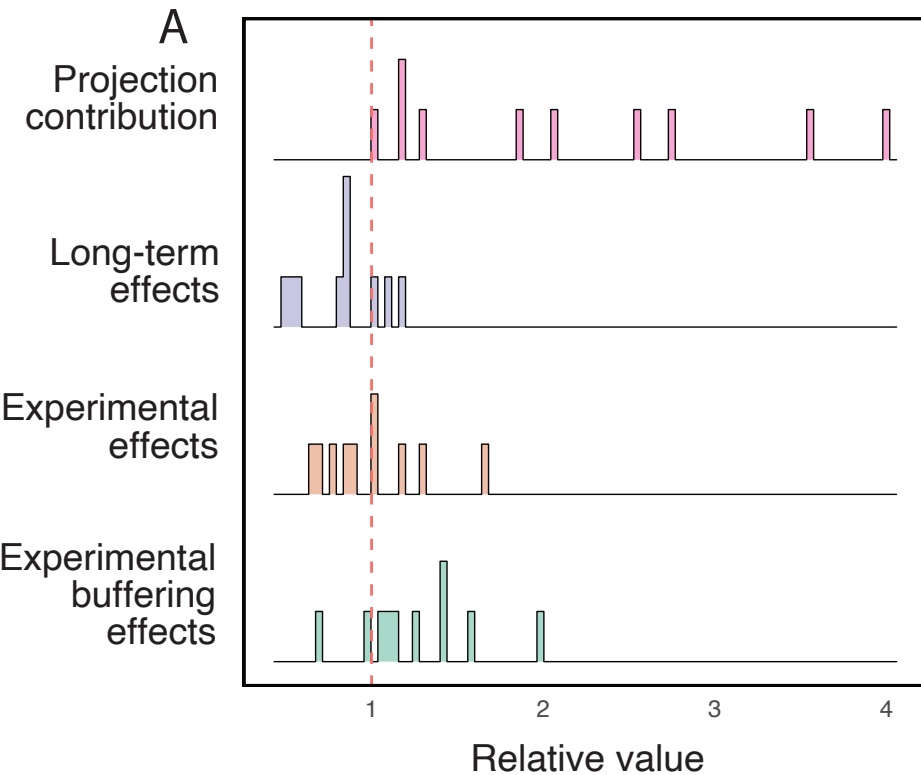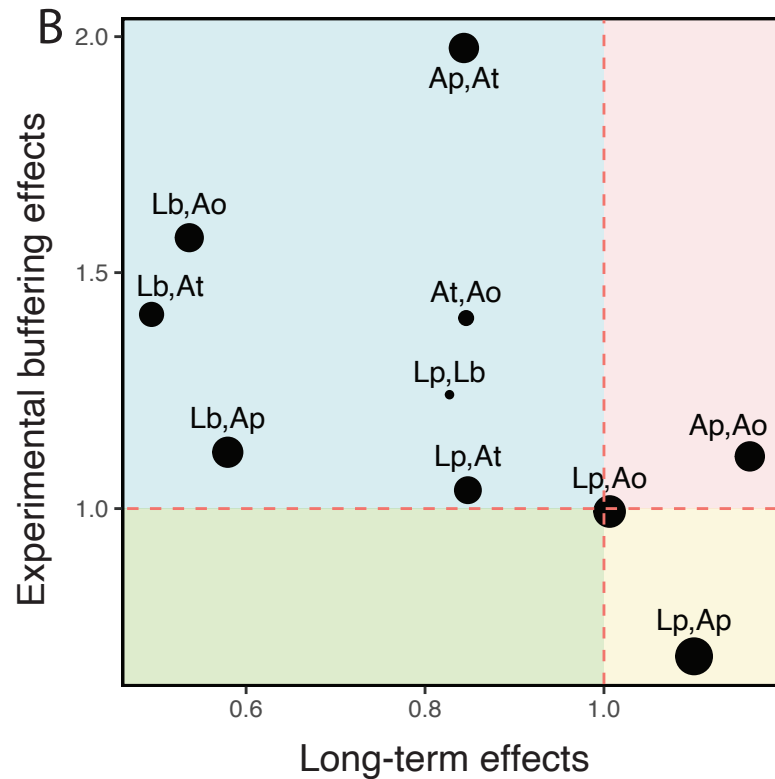

Supplement: S12 Fig — Compared to Fig 4 in the main text, here we classify a species statistically extinct in a trial if its relative abundance was less than 10% across all replicates (1% in Fig 4). (PDF) [file pcbi.1010630.s017.pdf]
