# Supplementary material for: Understanding the impact of third-party species on pairwise coexistence
Source: PLoS Comput Biol. 2022 Oct 24;18(10):e1010630. doi: 10.1371/journal.pcbi.1010630 (PMC9632822; doi:10.1371/journal.pcbi.1010630)
Supplement: S9 Fig — Panels A and C show the system-level effects on 45 pairs within a randomly-generated ten-species system characterized by negative-dominated and positive-dominated interactions, respectively (see Fig 2). Rows correspond to the projection distribution (PC(Z,S)), long-term effects (LE(Z,S)), short-term effects (SE(Z,S)), and buffering effects (BE(Z,S)), respectively. Note that each point in the distributions is a different pair within a system. For reference, the dashed line shows the value of 1. Recall that the x-axis corresponds to the change in probability of pairwise coexistence within the system. Panels B and D illustrate the cartographic representation for all corresponding pairs based on beneficial (LE(Z,S)>1) or detrimental (LE(Z,S)<1) long-term effects and beneficial (BE(Z,S)<1) or detrimental (BE(Z,S)<1) short-term effects. The size of points corresponds to the feasibility of pairs in isolation. The number of points in each region is annotated in gray. (PDF) [file pcbi.1010630.s014.pdf]

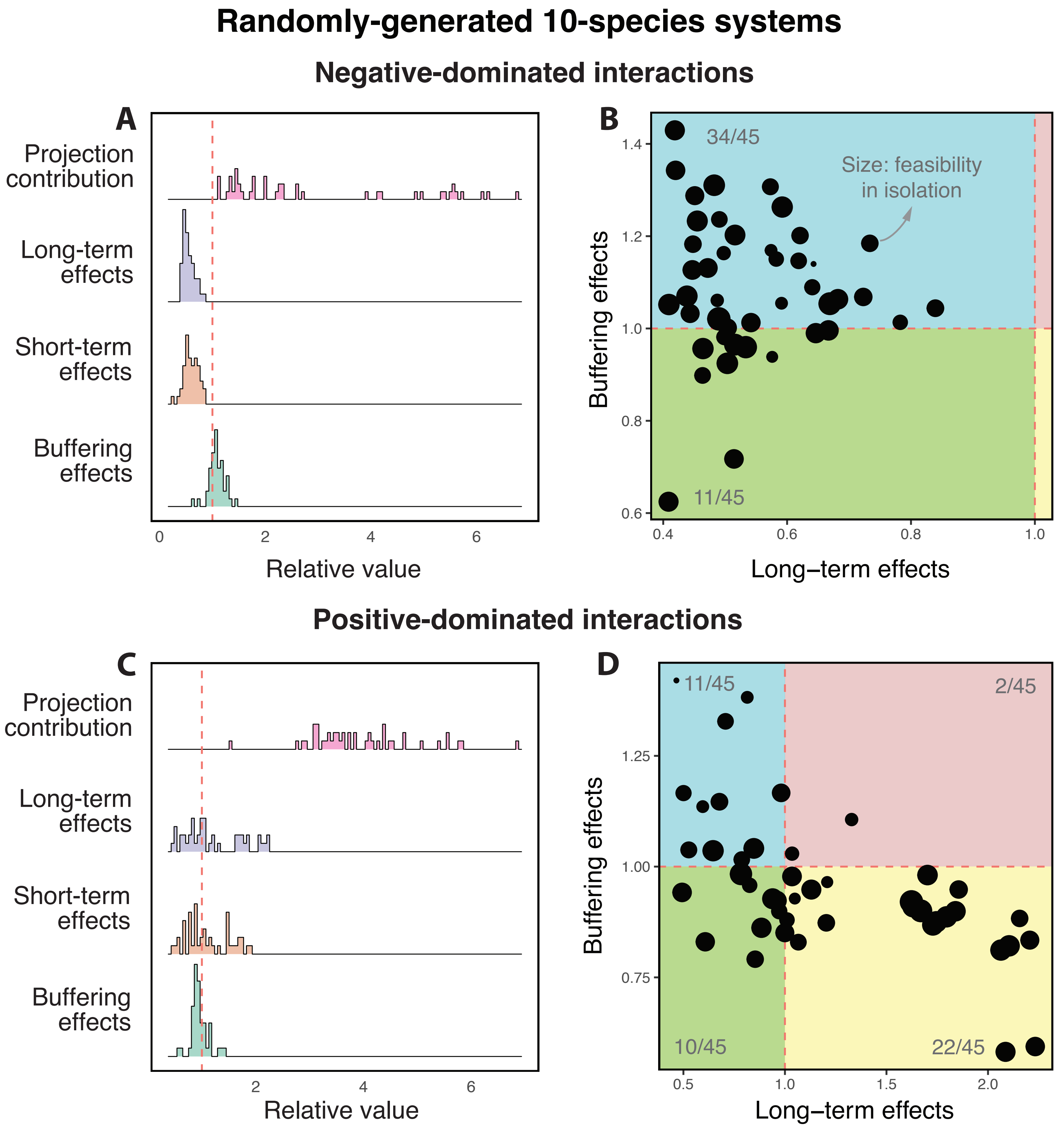

Randomly-generated 10-species systems
Negative-dominated interactions
A
Projection contribution
Long-term effects
Short-term effects
Buffering effects
0
2
4
6
Relative value
B
34/45
Size: feasibility in isolation
11/45
Buffering effects
1.4
1.2
1.0
0.8
0.6
0.4
0.6
0.8
1.0
Long−term effects
Positive-dominated interactions
C
Projection contribution
Long-term effects
Short-term effects
Buffering effects
2
4
6
Relative value
D
11/45
2/45
10/45
22/45
Buffering effects
1.25
1.00
0.75
0.5
1.0
1.5
2.0
Long−term effects
